# The Drug Repurposing for COVID-19 Clinical Trials Provide Very Effective Therapeutic Combinations: Lessons Learned From Major Clinical Studies

**DOI:** 10.3389/fphar.2021.704205

**Published:** 2021-11-18

**Authors:** Chiranjib Chakraborty, Ashish Ranjan Sharma, Manojit Bhattacharya, Govindasamy Agoramoorthy, Sang-Soo Lee

**Affiliations:** ^1^ Department of Biotechnology, School of Life Science and Biotechnology, Adamas University, Kolkata, India; ^2^ Institute for Skeletal Aging and Orthopedic Surgery, Hallym University-Chuncheon Sacred Heart Hospital, Chuncheon-si, South Korea; ^3^ Department of Zoology, Fakir Mohan University, Balasore, India; ^4^ College of Pharmacy and Health Care, Tajen University, Yanpu, Taiwan

**Keywords:** COVID-19, drug repurposing, treatment experience, future pandemics, clinical trials

## Abstract

SARS-CoV-2 has spread across the globe in no time. In the beginning, people suffered due to the absence of efficacious drugs required to treat severely ill patients. Nevertheless, still, there are no established therapeutic molecules against the SARS-CoV-2. Therefore, repurposing of the drugs started against SARS-CoV-2, due to which several drugs were approved for the treatment of COVID-19 patients. This paper reviewed the treatment regime for COVID-19 through drug repurposing from December 8, 2019 (the day when WHO recognized COVID-19 as a pandemic) until today. We have reviewed all the clinical trials from RECOVERY trials, ACTT-1 and ACTT-2 study group, and other major clinical trial platforms published in highly reputed journals such as NEJM, Lancet, etc. In addition to single-molecule therapy, several combination therapies were also evaluated to understand the treatment of COVID-19 from these significant clinical trials. To date, several lessons have been learned on the therapeutic outcomes for COVID-19. The paper also outlines the experiences gained during the repurposing of therapeutic molecules (hydroxychloroquine, ritonavir/ lopinavir, favipiravir, remdesivir, ivermectin, dexamethasone, camostatmesylate, and heparin), immunotherapeutic molecules (tocilizumab, mavrilimumab, baricitinib, and interferons), combination therapy, and convalescent plasma therapy to treat COVID-19 patients. We summarized that anti-viral therapeutic (remdesivir) and immunotherapeutic (tocilizumab, dexamethasone, and baricitinib) therapy showed some beneficial outcomes. Until March 2021, 4952 clinical trials have been registered in ClinicalTrials.gov toward the drug and vaccine development for COVID-19. More than 100 countries have participated in contributing to these clinical trials. Other than the registered clinical trials (medium to large-size), several small-size clinical trials have also been conducted from time to time to evaluate the treatment of COVID-19. Four molecules showed beneficial therapeutic to treat COVID-19 patients. The short-term repurposing of the existing drug may provide a successful outcome for COVID-19 patients. Therefore, more clinical trials can be initiated using potential anti-viral molecules by evaluating in different phases of clinical trials.

## 1 Introduction

The COVID-19 pandemic has created a global health crisis and massive mortality of over two million people worldwide. The disease began in December 2019 in Wuhan, China. Physicians and clinicians had initially described the conditions as “pneumonia of unknown etiology.” Subsequently, the disease spread around the world and affected nearly 187 countries. Initially, WHO declared it as a “health emergency” but finally declared it as a “pandemic” (Chauhan, 2020; Morens et al., 2020). The clinical symptoms associated with this disease include a broad-spectrum range of mild to severe respiratory problems. Depending on the kind of symptoms, patients can be categorized into three categories such as mild, moderate, and severe (Cascella et al., 2020). The fatality rate is estimated to be 2–5%. However, the fatality rate varies from country to country (Khafaie and Rahim, 2020). It was also observed that the mortality rate was high among elderly patients and patients with comorbidities such as diabetes, cardiovascular diseases, immune-suppressive diseases, and cancer (Kang and Jung, 2020; Rahim et al., 2020; Barbui et al., 2021).

**FIGURE 1:**
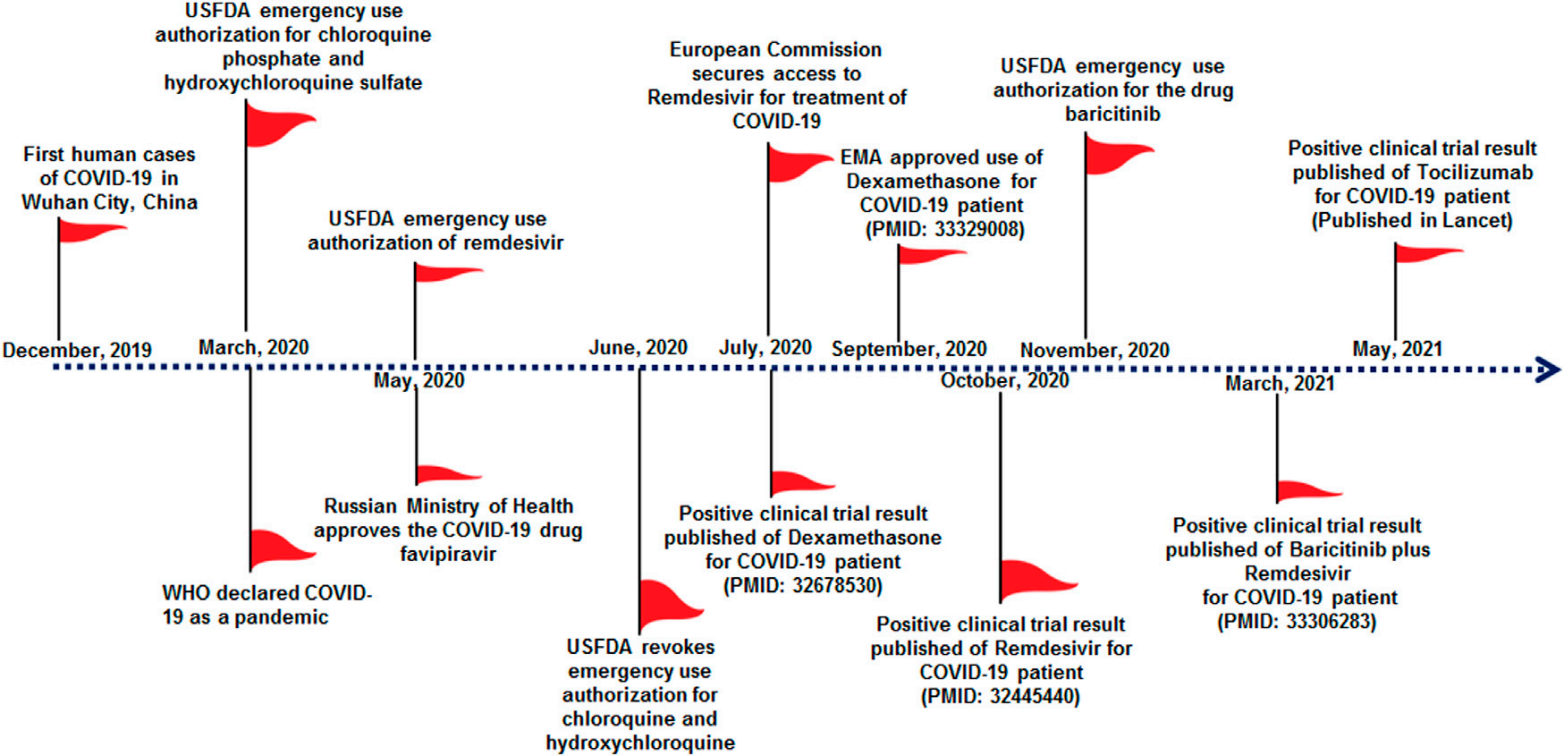
Significant milestones of COVID-19 therapeutic development in the previous 1 year, which show emergency approval of therapeutic molecules by different countries regulatory authorities for the treatment of COVID-19. This approval initiated several clinical trials for therapeutic repurposing of the drug for COVID-19 treatment, and significant clinical trials are listed in Table 2. Important milestones are listed in the timeline.

Initially, all physicians and clinicians have tried to find effective therapeutic molecules to treat this viral disease using repurposed drugs. Drug repurposing is an effective and rapid way to identify new use of existing drugs with a well-established safety profile (Pushpakom et al., 2019). Also, drug repurposing is a cost-effective way to treat disease outbreaks. Many drugs are already being successfully repurposed to treat various diseases (Kingsmore et al., 2020). Scientists have also started to explore therapeutics molecules against COVID-19 by utilizing repurposed molecules. Some existing anti-viral molecules are being applied to the *in vitro* system to understand their effectiveness against COVID-19 (Table 1). Some molecules demonstrated promising potential in pre-clinical trials. Several repurposed therapeutic molecules have received emergency approval from the USFDA and other regulatory authorities from different countries (Figure 1). Nonetheless, it appeared that most of the molecules are not useful to treat severe COVID-19 patients from time to time. Occasionally, some controversies developed for the therapeutic molecules related to their safety and efficacy, which were given emergency approval to treat COVID-19 patients (Gupta and Malviya, 2020; Mccreary and Meyer, 2021). At the same time, it was observed that several clinical trials were initiated to study the safety and effectiveness of several repurposed drugs for COVID-19 treatment. A recent paper reported that 3754 clinical trials had been completed. However, numerous clinical trial results have not been updated by organizations in the trial repositories (Rodgers et al., 2021). Therefore, transparency in the acquired data is urgently required to understand the safety and effectiveness of repurposed drugs. Meanwhile, there is an utmost urgency to distribute vaccines to fight against the pandemic. This review describes the status of the treatment of COVID-19 through drug repurposing. We have tried to evaluate the available data on repurposing drugs from the beginning of their trials, including the lessons learned from the experience from using various therapeutic molecules to treat the COVID-19 patients like hydroxychloroquine, ritonavir/lopinavir, favipiravir, remdesivir, ivermectin, dexamethasone, camostatmesylate, tocilizumab, mavrilimumab, baricitinib, and interferons (IFN).

**TABLE 1:** List of different repurposed therapeutic molecules for COVID-19.

Sl no	Repurposed therapeutic molecules	Previously approved diseases for the treatment	Remark
1.	Chloroquine phosphate and hydroxychloroquine sulfate	Treatment for malaria, amoebic dysentery	Emergency approval was given all repurposed therapeutic. But emergency arrival of chloroquine and hydroxychloroquine was revoked due to cardiac toxicity. All repurposed therapeutic clinical trials result was recorded in Table 2 (tested for COVID-19)
2.	Azithromycin	Treatment for chest infections (pneumonia), infections of nose and throat, skin infections, Lyme disease, and some sexually transmitted infections	
3.	Lopinavir-Ritonavir	Treatment and prevention of HIV/AIDS	
4.	Favipiravir	Treatment for influenza	
5.	Remdesivir	Treatment for Ebola virus disease and Marburg virus infections	
6.	Ivermectin	Treatment for parasite infestations (head lice, scabies, river blindness, strongyloidiasis, trichuriasis, ascariasis, and lymphatic filariasis)	
7.	Dexamethasone	Treatment for skin diseases, severe allergies, asthma etc.	
8.	Tocilizumab	Treatment of rheumatoid arthritis and systemic juvenile idiopathic arthritis	
9.	Mavrilimumab	Treatment of rheumatoid arthritis	
10.	Baricitinib	Treatment of rheumatoid arthritis	

## 2 Lessons Learned from Therapeutic Molecules Development

Several drugs that have been approved for other viral diseases are being used in treating COVID-19 patients. They are hydroxychloroquine, ritonavir/lopinavir, favipiravir, remdesivir, ivermectin, dexamethasone, camostatmesylate, etc. (Table 2).

**TABLE 2:** Lessons learned from a clinical trial for therapeutic repurposing against COVID-19.

No	Drug molecules	Lessons learned from clinical trial	Clinical trial No	References
1.	Hydroxychloroquine with or without Azithromycin	The use of hydroxychloroquine alone or with azithromycin did not shows improvement of the clinical status of hospitalized COVID-19 patients with mild-to-moderate symptoms, compared to standard care	Clinical trial no. NCT04322123 from ClinicalTrials.gov	Cavalcanti et al. (2020)
2.	Hydroxychloroquine, azithromycin, or both these combination	The use of hydroxychloroquine, azithromycin, or both these drugs are not significantly associated with mortality in- hospitalized COVID-19 patients	—	Rosenberg et al. (2020)
3.	Hydroxychloroquine	Hydroxychloroquine did not considerably improvement of clinical status of hospitalized COVID-19 patients at day 14	Clinical trial no.NCT04332991 from ClinicalTrials.gov	Self et al. (2020)
4.	Lopinavir-Ritonavir	Lopinavir–ritonavir shows no benefit in the treatment for the hospitalized adult COVID-19 patients with severe symptoms	Clinical trial no ChiCTR2000029308. from Chinese Clinical Trial Registry	Cao et al. (2020)
5.	Lopinavir-ritonavir	No advantage was noted for use of lopinavir-ritonavir among hospitalized COVID-19 patients.The trial did not recommend the use of lopinavir-ritonavir for treatment of COVID-19 patients (RECOVERY clinical trial platform)	Clinical trial noNCT04381936 from ClinicalTrials.gov	Horby et al. (2020a)
6.	Favipiravir	No additional benefit was found using favipiravir (up to 100 μM concentration)	Chinese Clinical Trial Registry no. ChiCTR 2000029544	Lou et al. (2021)
7.	Remdesivir	From a randomized, double blind clinical trials with 1,062 patients, remdesivir help to shorten the time to recovery in hospitalized COVID-19 adults patients compare to hospitalized COVID-19 patients who are taking placebo (ACTT-1 Study Group)	Clinical trial no.NCT04280705 from ClinicalTrials.gov	Beigel et al. (2020)
8.	Remdesivir	Remdesivir did not shows major clinical benefits among hospitalized severe COVID-19 patients	Clinical trial no NCT04257656 from ClinicalTrials.gov	Wang et al. (2020b)
9.	Ivermectin	Non-severe COVID-19 patients treated with ivermectin recovered earlier from anosmia/hyposmia	Clinical trial no. NCT04390022 from ClinicalTrials.gov	Chaccour et al. (2021)
10.	Ivermectin	The used doses of ivermectin (IVM 200 μg/kg, single dose) does not affect severe COVID-19 patients and the drug has no impact in decreasing the COVID-19 related mobility on	Approved by Ethics Committee, Hospital Clinic in Barcelona, Spain (HCB/2020/0475)	Camprubí et al. (2020)
11.	Dexamethasone	The use of this therapeutic corticosteroid resulted in lower 28-day mortality among the hospitalized with COVID-19 patients who were receiving either oxygen alone or invasive mechanical ventilation support (RECOVERY clinical trial platform)	Clinical trial no. NCT04381936 from ClinicalTrials.gov	Horby et al. (2021)
12.	Tocilizumab	COVID-19 patients who received tocilizumab had fewer severe infections compare to patients who has given placebo drug	Clinical trial no.NCT04356937, NCT04381936 from ClinicalTrials.gov	Stone et al. (2020)
13.	Tocilizumab	Tocilizumab shows beneficial effect in hospitalised COVID-19 patient (RECOVERY clinical trial platform)	Clinical trial no. NCT04381936 from ClinicalTrials.gov	Abani et al. (2021)
14.	Tocilizumab	This Indian clinical trial did not support the routine use of this drug among the hospitalized patients with moderate to severe symptoms	Clinical trial no.CTRI/2020/05/025369 from Indian clinical trials registry	Soin et al. (2021)
15.	Mavrilimumab	Mavrilimumab improved clinical outcomes severe COVID-19 compared with standard care. A further multicenter randomized controlled trial is ongoing	Clinical trial no. NCT04397497 from ClinicalTrials.gov	De Luca et al. (2020a), De Luca et al. (2020b)
16.	Baricitinib	Baricitinib may speeds up viral clearance. It may decrease the Intensive Care Unit (ICU) admission rate and fatality rate. Several clinical trials are undergoing to understand the advantage of baricitinib for COVID-19 treatment	Clinical trial no. NCT04373044 from ClinicalTrials.gov; Clinical trial no.NCT04421027 from ClinicalTrials.gov; Clinical trial no.NCT04640168 from ClinicalTrials.gov	Cantini et al., (2020), Richardson et al. (2020)
17.	Baricitinib with remdesivir	From a randomized clinical trial with 1033 patients, this study shows the better therapeutic outcome of this combination drug for COVID-19 hospitalised patient compare to remdesivir (Adaptive COVID-19 Treatment Trial 2 study group)	ClinicalTrials.gov; NCT04401579	Kalil et al. (2021)

### 2.1 Chloroquine and Hydroxychloroquine

Anti-parasitic drugs such as chloroquine and hydroxychloroquine were repurposed for the treatment of the COVID-19. Chloroquine has been used for over 70 years worldwide, and it is part of the essential medicines as per WHO documentation (Cortegiani et al., 2020). This medication has immunosuppressive effects. Due to this property, chloroquine has been used successfully against several autoimmune diseases such as lupus and rheumatoid arthritis (Browning, 2014). Since the late 1960s, this drug’s anti-viral properties were explored, and the anti-viral activity effect was established (Touret and De Lamballerie, 2020). On the other hand, the side effects of chloroquine on the central nervous system (CNS) were also recorded (Phillips-Howard and Ter Kuile, 1995).

Similarly, hydroxychloroquine is also helpful in treating rheumatoid arthritis, lupus, and autoimmune diseases (Nirk et al., 2020). It was used in the treatment of COVID-19 patients. In this direction, USFDA gave emergency approval to hydroxychloroquine and chloroquine in March 2020 (FDA, 2020). Likewise, some other counties also provided the emergency approval of these molecules to treat COVID-19 patients (Table 3) (Desk, 2020). Several clinical trials were also undertaken to understand the effectiveness of this drug in treating COVID-19 patients. In some clinical trials, hydroxychloroquine was used to treat the COVID-19 patients alone or with a macrolide antibiotic, azithromycin. Mokhtari et al. performed a clinical trial with 28,759 mild COVID-19 patients where they reported reduced death or hospitalization among patients. In addition, no profound adverse effect was noted by this study (Mokhtari et al., 2021). However, the study did not recruit severe COVID-19 patients for the trial.

**TABLE 3:** List of emergency approval or withdrawal of various therapeutic molecules to treat COVID-19 patients.

Sl no	Therapeutic molecule	Name of the countries	Approval authorities/Regulatory authorities	References
1.	Chloroquine phosphate and hydroxychloroquine sulfate	United States	USFDA	USFDA (2020)
2.	Chloroquine phosphate and hydroxychloroquine sulfate	India	DCGI	Desk (2020)
3.	Chloroquine phosphate and hydroxychloroquine sulfate	United States	USFDA revoked the approval	Hinton, (2020a)
4.	Remdesivir	United States	USFDA	Hinton (2020b)
5.	Remdesivir	Japan	PMDA	Yasuhiro (2020)
6.	Remdesivir	India	DCGI	Welfare (2020b)
7.	Remdesivir	Singapore	HSA	Authority, (2020)
8.	Remdesivir	South Korea	MFDS	Safety, Ministry of Drug and Food (2020)
9.	Remdesivir	Taiwan	TFDA	Welfare (2020a)
10.	Remdesivir	Australia	TGA	Administration (2020a)
11.	Remdesivir	European Union	European Medicines Agency (EMA)	Commission (2020)
12.	Favipiravir	China	NMPA	Highlights (2020)
13.	Favipiravir	India	DCGI	Ltd (2020)
14.	Favipiravir	Russia	Ministry of Health (Minzdrav)	RDIF (2020)
15.	Dexamethasone	India	DCGI	Organisation (2020)
16.	Baricitinib	United States	USFDA	Administration (2020b)

Similar to this study, another randomized clinical trial was performed with 667 participants in Brazil using hydroxychloroquine alone or along with azithromycin. However, the trial reported no improvement or benefit of this drug during the treatment of mild-to-moderate COVID-19 patients (clinical trial no. NCT04322123 from ClinicalTrials.gov) (Cavalcanti et al., 2020). Another study for its efficacy was conducted in New York State using 1438 hospitalized COVID-19 patients. The study also found no benefit with hydroxychloroquine alone or with azithromycin in treating COVID-19 patients (Rosenberg et al., 2020).

Conversely, the cardiac toxicity of hydroxychloroquine cannot be overlooked if COVID-19 patients have pre-existing cardiac complications. Due to this adverse effect, on June 14, 2020, these drugs were withdrawn for clinical applications (Hinton, 2020a). Later, in December 2020, an article was published in JAMA where researchers observed from a randomized clinical trial (NCT04332991; ClinicalTrials.gov) that hydroxychloroquine did not show any clinical benefits for the hospitalized adult COVID-19 patient’s respiratory illness. Instead, the study compared it with placebo (Self et al., 2020).

### 2.2 Lopinavir/Ritonavir

Lopinavir (ABT-378) and ritonavir (ABT-538) are protease inhibitors (PI) that are used as a medication against HIV/AIDS. These two molecules are structurally related PI (Cvetkovic and Goa, 2003). Both demonstrated *in vitro* anti-viral effects against SARS coronavirus (Chen et al., 2004). The combination of ritonavir/lopinavir was found effective *in vitro* and in an animal model of MERS-CoV (Arabi et al., 2018; Yao et al., 2020). Ratia et al. observed that using a fixed-dose combination of ritonavir/lopinavir, the main protease of SARS-CoV-1 might be blocked. In this case, ritonavir may boost lopinavir concentrations, and ritonavir acts as a potent CYP3A4 inhibitor (Ratia et al., 2008). This anti-viral combination therapy was repurposed for the COVID-19 patients, and several clinical trials were conducted. A randomized clinical trial was carried out for 199 COVID-19 patients at Jin Yin-tan Hospital in Wuhan, China. The study utilized combination therapy of ritonavir/lopinavir, but the results showed no significant benefits for severe adult COVID-19 patients and did not reduce mortality (clinical trial any ChiCTR2000029308 Chinese Clinical Trial Registry). The study concluded that the drug regimen could not be regarded as a valuable and life-saving combination therapy. Also, the study was discontinued for 13 patients due to adverse effects in the patients (Cao et al., 2020). The recovery trial group performed another randomized, open-label, controlled trial. In this trial, lopinavir-ritonavir combination treatment was received by 1616 people, and usual care was provided to 3424 patients. The study concluded no advantage for using lopinavir/ritonavir among hospitalized COVID-19 patients and not associated with decreased mortality in 28 days during their hospital stay. At the same time, lopinavir/ritonavir therapy was not found related to the possibility of progressing to persistent mechanical ventilation or death (clinical trial noNCT04381936 from ClinicalTrials.gov) (Horby et al., 2020a).

### 2.3 Favipiravir

The anti-influenza medicine favipiravir (T-705) was repurposed for COVID-19 patents. This drug was approved for the treatment of influenza in 2014 and was developed in Japan. This drug inhibits the viral RNA-dependent RNA polymerase (RdRp) (Furuta et al., 2017). China was the first to announce that favipiravir had shown good clinical efficacy against COVID-19 (Xinhuanet, 2020). However, Chen et al. described no confirmative evidence on its advantage to COVID-19 patients (Chen PJ. et al., 2020). In a recent study, favipiravir and other molecules such as baloxavir and marboxil were evaluated at the Zhejiang University’s School of Medicine. Researchers reported no extra benefit of favipiravir under the trial dosages against COVID-19 patients (Chinese Clinical Trial Registry no. ChiCTR 2000029544) (Lou et al., 2021).

### 2.4 Remdesivir

Remdesivir (GS-5734) is another anti-viral drug that got emergency approval from the regulatory authorities of different countries for COVID-19 patients (Saha et al., 2020a; 2021). This drug is considered an essential medicine among the repurposing drugs against SARS-CoV-2 (Españo et al., 2021). The drug shows therapeutic efficacy in viruses such as Ebola (Warren et al., 2016; Siegel et al., 2017), Nipah (Jordan et al., 2018; Lo et al., 2019), SARS-CoV-2, MERS CoV, and SARS-CoV (Malin et al., 2020). Remdesivir shows *in vitro* activity by binding to the RNA-dependent RNA polymerase (RdRp), and it acts as a terminator for RNA elongation (Wang M. et al., 2020). Recent studies show that it may be helpful for clinical improvement against SARS-CoV-2. Beigel et al. have illustrated in their first clinical trial that it may be better than placebo as it shortened the time to recovery in hospitalized adults with COVID-19. This randomized trial recruited 1062 patients, and 541 received remdesivir while 521 received placebo (clinical trial no. NCT04280705 from ClinicalTrials.gov) (Beigel et al., 2020). It was observed that the remdesivir does not show any significant advantage at day 28 with mild to moderately symptomatic COVID-19 population, and this group of patients does not need any respiratory support. At the same time, it was also observed that the remdesivir benefits the patients with hyper-inflammation requiring supplemental oxygen. In this case, it reduces the risk of progression and shortens the recovery time if illness is detected early (≤10 days) (Young et al., 2021). In another study, Wang et al. conducted a double-blind, randomized, multicenter placebo-controlled trial in Hubei, China across 10 hospitals and 237 patients were enrolled. Of them, 158 COVID-19 patients received remdesivir while 79 received placebo. The study concluded that the drug was not related to statistically considerable clinical benefits for hospitalized severe COVID-19 patients (clinical trial no NCT04257656 from ClinicalTrials.gov) (Wang Y. et al., 2020).

### 2.5 Ivermectin

Another anti-parasitic drug, ivermectin was tried for the treatment of COVID-19 patients since it showed anti-viral properties (Heidary and Gharebaghi, 2020; Kory et al., 2021). *In vitro* study showed beneficial effects of ivermectin against SARS-CoV-2 including the inhibition of the virus’s replication (Caly et al., 2020). However, it was noted that the drug could create toxic effects in COVID-19 patients since pharmacokinetic issues were documented (Jermain et al., 2020; Momekov and Momekova, 2020; Peña‐Silva et al., 2020). A placebo-controlled, double-blind, randomized pilot clinical trial (Phase-II) was conducted using non-severe COVID-19 patients where patients were treated with the ivermectin and placebo drug. After the trial, the study concluded that non-severe COVID-19 patients treated with ivermectin recovered at a rapid rate from anosmia/hyposmia. However, the small size of this randomized clinical trial may not be enough to regard it as a life-saving therapy.

Along with the recovery, lower IgG titers, a lower viral load, and a reduction in cough were noted in ivermectin-treated COVID-19 patients (clinical trial no. NCT04390022 from ClinicalTrials.gov) (Chaccour et al., 2021). Recently a study was conducted with severe COVID-19 patients in Spain. In that study, two groups were created: IVM group, patients treated with ivermectin (*n* = 13)) and non-IVM group, patients not treated with ivermectin (*n* = 13)). The patients were treated with a dose of 200 μg/kg ivermectin per single shot. The study confirmed that ivermectin did not affect severe COVID-19 patients and concluded that the drug had no impact on decreasing the COVID-19-related morbidity (Camprubí et al., 2020).

### 2.6 Dexamethasone

Dexamethasone is an old therapeutic molecule widely used to restrain allergic inflammations (Giles et al., 2018; Selvaraj et al., 2020), and it is in use since the 1960s. It is the first drug that showed life-saving efficacy in COVID-19 patients (Lammers et al., 2020). Several clinical trials have been performed to understand the efficacy of this drug for COVID-19 patients. The RECOVERY collaborative group recently conducted a clinical trial to evaluate the dexamethasone with a 6 mg dose daily for 10 days to more than 6000 hospitalized COVID-19 patients. Among them, 4321 received the usual care while 2104 patients received dexamethasone. The study found that the therapeutic corticosteroid lowered the 28-day mortality among the hospitalized patients who received either oxygen alone or invasive mechanical ventilation support. Nevertheless, there was no effect on those patients who received respiratory support (clinical trial no. NCT04381936 from ClinicalTrials.gov) (Horby et al., 2021). This was a randomized clinical trial. However, European Medical Association (EMA) approved dexamethasone to treat COVID-19 patients (Gozzo et al., 2020). It is the first drug that significantly helped in the recovery and survival of COVID-19 patients in a randomized controlled trial. Moreover, the drug is highly economical and widely available in the market (Ledford, 2020).

### 2.7 Camostatmesylate

Camostat (NI-03) is a serine protease inhibitor TMPRSS2 and was approved for reflux esophagitis and pancreatitis in Japan. SARS-CoV-2 utilizes TMPRSS2 protease to bind with the ACE2 receptor for entering the host cell (Chakraborty et al., 2021). A study found that camostat can inhibit the SARS-CoV-2 entry, especially in human epithelial cells (Hoffmann et al., 2020a; Hoffmann et al., 2020b). However, several randomized clinical trials are currently going on to understand these therapeutic molecules’ safety and efficacy on COVID-19 patients (clinical trial no. NCT04321096, clinical trial no. NCT04625114, clinical trial no. NCT04608266 from ClinicalTrials.gov).

### 2.8 Heparin

Heparin, especially a low molecular weight (LMW) heparin, helps to treat the COVID-19 patients. This drug might help to halt cytokine storms in severe COVID-19 patients. In addition, due to its anti-viral and anti-inflammatory properties, it may assist in reducing mortality in COVID-19 patients (Barcellona et al., 2021). Some clinical trials reported that the molecule could be effective for the treatment of COVID-19 patients. A multicentric clinical trial was conducted in 17 hospitals in Spain with 2075 hospitalized COVID-19 patients. Among them, 1447 patients recovered, while 301 patients died (Ayerbe et al., 2020). However, more randomized clinical trials are required to understand the efficacy of heparin in COVID-19 patients. Another study reported that heparin, especially LMW heparin, lowers the mortality rate in patients with high D-dimer levels (Tang et al., 2020a). In some situations, the COVID-19 patients are associated with poor prognoses, such as abnormal coagulation factors. In this case, the patients with elevated D-dimer levels (abnormal coagulation parameter) are associated with a poor prognosis of the diseases. However, Tang et al. have reported that heparin (especially LMW heparin) improves the prediction of the disease in such conditions (Tang et al., 2020b).

## 3 Lessons Learned from Immunotherapeutic Molecules Development

Immune suppressors and modulators have been considered critically significant therapeutic candidates to treat severe COVID-19 patients with cytokine storms. The cytokine storm has a devastating consequence on immune dysregulation in patients (Fajgenbaum and June 2020; Mehta et al., 2020). This condition can cause organ failure and increase the fatality rate (Peng et al., 2021). However, several immunotherapeutic molecules are repurposed to treat the cytokine storm among COVID-19 patients.

### 3.1 Tocilizumab

It is a humanized monoclonal antibody (hmAB), also called tocilizumab. It can act against the interleukin-6 receptor (IL-6R) in membrane-bound and soluble forms (Saha et al., 2020b). This molecule can be used to treat cytokine syndrome disease (Kotch et al., 2019). A clinical trial concluded that tocilizumab could treat severe COVID-19 patients to reduce mortality effectively. However, the study cautioned to be a preliminary one. Therefore, more clinical evidence is required to prove this molecule’s effectiveness in treating severe COVID-19 patients (Xu et al., 2020). A recent study was conducted with 544 (out of 1351 patients) severe COVID-19 patients with pneumonia. In this trial, 13 patients were treated with tocilizumab. Among them, 6 received tocilizumab intravenously and 7 subcutaneously. The study concluded that whether tocilizumab, administered subcutaneously or intravenously, might reduce the risk of death or mechanical ventilation in severe COVID-19 patients with pneumonia (Guaraldi et al., 2020). Another clinical study was conducted with 51 patients where 23 did not receive tocilizumab therapy while 28 patients received tocilizumab treatment. It concluded that tocilizumab was linked with a considerably shorter duration of vasopressor support in severe COVID-19 patients. However, researchers have remarked that the finding needs corroboration with the ongoing clinical trials of COVID-19 patients using tocilizumab (Kewan et al., 2020).

A recent randomized clinical trial study was published in NEJM, which was conducted with 243 patients. This study has shown that COVID-19 patients who received this drug had fewer severe infections than patients who were given a placebo drug. The study concluded that the molecule was not efficient for preventing the death of moderately ill hospitalized patients with COVID-19. However, they also concluded that a few advantages or damage could not be ruled out (clinical trial no. NCT04356937 from ClinicalTrials.gov) (Stone et al., 2020). Furthermore, a randomized clinical trial was conducted in India using 180 COVID-19 patients. Patients were divided into two groups (standard care group; *n* = 90) and the other (tocilizumab group; *n* = 90). The study did not support the routine use of hmAB based drugs among the hospitalized patients with moderate to severe symptoms (Clinical trial no. CTRI/2020/05/025369 from Indian clinical trials registry) (Soin et al., 2021). Therefore, there is a controversy about the role of tocilizumab in the treatment of severe COVID-19 patients (Mccreary and Meyer, 2021). However, RECOVERY group clinical trials have solved the debate by showing beneficial effects of tocilizumab in hospitalized COVID-19 patients (Clinical trial no. NCT04381936) (Abani et al., 2021).

### 3.2 Mavrilimumab

Mavrilimumab (CAM-3001) is an hmAB that helps to inhibit a receptor, GMCSFR. Therefore, it is also called an anti-GM-CSFR monoclonal antibody (Nair et al., 2012). In a single-center clinical trial, De Luca and colleagues conducted a clinical study with COVID-19 patients. Patients were divided into two groups: the first group had 13 non-mechanically ventilated patients receiving mavrilimumab, and the other groups had 26 patients receiving standard care with routine care and non-mechanical ventilation. The second group was regarded as the control group. This study concluded that the monoclonal antibody treatment was associated with improved clinical outcomes for severe COVID-19 patients with systemic hyper-inflammation and pneumonia (De Luca et al., 2020a). Pourhoseingholi et al. tried to affirm this study by asking for statistical analysis of the trial to get the conclusion (Pourhoseingholi et al., 2020). However, De Luca and colleagues stated that a further multicenter randomized controlled trial is ongoing using mavrilimumab to support their hypothesis, and results are awaited (clinical trial no. NCT04397497 from ClinicalTrials.gov) (De Luca et al., 2020b).

### 3.3 Baricitinib

This therapeutic small-molecule is a Janus kinase (JAK) inhibitor (JAK1/2 inhibitor), and it has been approved for the treatment of rheumatoid arthritis (Schwartz et al., 2017). Richardson concluded that baricitinib might be a potential candidate to treat COVID-19 (Richardson et al., 2020). It was observed that this therapeutic molecule might decrease the intensive care unit admission and fatality rate. Baricitinib speeds up the viral clearance and augments patients’ discharge rates compared to COVID-19 patients who are having standard-of-care. COVID-19 patients with moderate pneumonia were treated with baricitinib (Cantini et al., 2020). Several randomized clinical trials are undergoing to understand the advantage of baricitinib for COVID-19 treatment (clinical trial NCT04373044, NCT04421027, and NCT04640168 [ClinicalTrials.gov]). However, a recent randomized clinical trial with 1033 patients showed a better therapeutic outcome of combined therapy of baricitinib with remdesivir for COVID-19 hospitalized patients compared to only remdesivir (ACTT-2 Study Group) (NCT04401579) (Kalil et al., 2021).

### 3.4 Interferons Therapy

Few clinical studies have noted that IFNs alone or with other agents might help to treat mild to moderate COVID-19 patients (Fu et al., 2020; Hung et al., 2020). Some studies observed ambiguous evidence for the role of IFN in COVID-19 progression. However, several clinical trials are undergoing to evaluate the advantages of IFN treatment and confirm whether IFN therapy will be beneficial.

## 4 Combination Therapy or Multidrug Therapy for COVID-19 Patients

Recently combination therapy has shown some prospective on COVID-19 patients. Some study indicates that combination therapy or multidrug therapy for COVID-19 outpatients might reduce hospitalization and death by ∼85%. In some cases, combination therapy displays outstanding results in the treatment for COVID-19 patients. However, in severe COVID-19 patients, several devastating conditions were observed, such as cytokine storm, viral-mediated organ damage, and thrombosis. Therefore, McCullough et al. have concluded that combination/multidrug therapy is a significant criterion for treating COVID-19 patients with life-threatening conditions (McCullough et al., 2020).

As discussed earlier, a randomized clinical trial of combined treatment of baricitinib with remdisivir to COVID-19 hospitalized patients demonstrated better outcomes than treatment of remdesivir alone (ACTT-2 Study Group) (NCT04401579) (Kalil et al., 2021). In another clinical trial, 1694 COVID-19 patients were treated with two drugs (remdesivir and dexamethasone) along with the standard-of-care (SOC). The result shows a reduction in 30-day mortality (Benfield et al., 2021). Similarly, another randomized clinical trial was performed for COVID-19 patients using the combination therapy of etesevimab and bamlanivimab. The study also tried to understand the effect of monotherapy of bamlanivimab. Thus, the study attempted to analyze the impact of both the therapy (monotherapy and combination therapy) of COVID-19 patients with a mild to moderate viral load. On day 11, the study noted a statistically significant reduction in viral load among the patients who received combination therapy (bamlanivimab and etesevimab) compared with placebo. No significant difference was observed in the decrease in the patients’ viral load with monotherapy (Gottlieb et al., 2021).

Procter et al. have evaluated the effects of the combination therapy on 922 outpatients from March to September 2020. In their treatment, two therapeutic agents (one anti-viral molecule and one antibiotic) were used. The study used anti-viral molecules like ivermectin, hydroxychloroquine, and zinc. The three antibiotics that were used are doxycycline, azithromycin, and ceftriaxone (Procter et al., 2020). The study concluded that multidrug/combination therapy treatment is more feasible and safer for early symptomatic patients, treated either at home or not hospitalized. In addition, multidrug/combination therapy was associated with reduced death and low hospitalization rates (Procter et al., 2021). A combination therapy (triple therapy) of zinc, azithromycin, and low-dose hydroxychloroquine was evaluated in 141 COVID-19 patients. The study observed no cardiac side effects and significantly fewer hospitalizations in the study group (Derwand et al., 2020). Nevertheless, more clinical research is required to evaluate the effect of different combination therapy/multidrug for COVID-19 patients.

## 5 Lessons Learned from Convalescent Plasma from Recovered Patients

It was stated that convalescent plasma from recovered patients might help in treating COVID-19 patients (Duan et al., 2020; Shen et al., 2020; Tanne, 2020). However, several clinical trials are ongoing with convalescent plasma from recovered patients, and these studies need to be peer-reviewed after completion.

## 6 Lessons Learned from Neutralizing Antibodies Against SARS-CoV-2

Neutralizing antibodies (nAbs) can be transferred to people to treat disease or prevent disease. The nAbs can be used before and after SARS-CoV-2 infection (Jiang et al., 2020). Several studies were performed to developed human nAbs. During the development, researchers used the SARS-CoV-2 Spike glycoprotein or their part such as SARS-CoV-2 RBD, SARS-CoV-2 NTD, etc. Among the neutralizing antibodies, some are in the pre-clinical stage, and some are in the clinical trial phase. In this direction, Ju et al. isolated a human nAbs named P2C-1F11 and P2B-2F6, targeting SARS-CoV-2 RBD (Ju et al., 2020). At the same time, Rogers et al. isolated human nAbs termed CC6.29, CC6.30, and CC12.1 that target SARS-CoV-2 RBD (Rogers et al., 2020). Shi et al. isolated another human nAbs named CA1 and CB6, targeting antigen SARS-CoV-2 RBD (Shi et al., 2020). Some developed nAbs are in the clinical trial phase. FDA has approved monoclonal antibodies for the treatment of patients with COVID-19 (Coronavirus, 2021). Recently a Phase II/III trial is evaluating the safety and efficacy of combination therapy of human nAb (REGN10933 + REGN10987). The injectable was administrated through the intravenous or subcutaneous route as a single dose. This study is a randomized, placebo-controlled study where 6420 participants were recruited (Clinical trial no: NCT04425629; ClinicalTrials.gov). In November 2020, US-FDA provided emergency approval for the use of the two neutralizing antibodies such as imdevimab (REGN10987) and casirivimab (REGN10933) (Du et al., 2021; Hurt and Wheatley, 2021). Presently, imdevimab and casirivimab combination (REGN-COV2) is entitled as “antibody cocktail.” Another antibody named bamlanivimab (LY-CoV555) is also called LY3819253. Eli Lilly collaborated with AbCellera (a biotechnology company based in British Columbia) to develop this antibody (Sun and Ho, 2020). Companies have started a human clinical trial. In this direction, they have performed clinical trials such as the BLAZE-1 trial, ACTIV-2 trial, and ACTIV-3 trial. NIH sponsored the ACTIV-2 trial. The USFDA granted the antibody a EUA in November 2020 for the treatment of mild-to-moderate COVID-19 patients. However, it was reported that the EUA was revoked in April 2021. Nevertheless, the USFDA granted a EUA to administer two antibodies together (bamlanivimab and etesevimab) to treat mild-to-moderate COVID-19 patients in February 2021. A phase III trial was performed among 1035 patients using this drug combination (bamlanivimab and etesevimab) or placebo. The study found lower cases of death and hospitalization among COVID-19 patients compared to placebo (Dougan et al., 2021). Simultaneously, Gottlieb et al. performed a clinical trial using combination therapy with etesevimab, bamlanivimab, and bamlanivimab monotherapy. The trial tried to understand the viral load in mild to moderate COVID-19 patients in a randomized manner. However, the study found a significant reduction in viral load at day 11 among the patients treated with etesevimab and bamlanivimab. It was also noted that there was no significant reduction in viral load for the patients who were treated with bamlanivimab monotherapy (Gottlieb et al., 2021).

Recently emerging variants have evolved, which are significant concerns for different countries (Chakraborty et al., 2021a; Chakraborty et al., 2021b; Chakraborty et al., 2021c). Tea et al. evaluated nAbs response against the variants and found the antibody evasion. This study may help in SARS-CoV-2 antibody responses and vaccine monitoring (Tea et al., 2021). However, more clinical trials are needed to understand the safety and efficacy profile of nAbs against COVID-19. In addition, cost-effective and efficient nAbs are also required, having more efficacy against COVID-19.

## 7 Symptomatic Treatment is Necessary

Several COVID-19 patients show symptoms like fever with headache. In such cases, symptomatic treatment is required, and the drug of choice is acetaminophen (paracetamol) (Belvis, 2020). Several times in COVID-19 patients, the pneumonia-associated respiratory disorder is observed (Chakraborty et al., 2020). Therefore, several antibiotics can be used to treat pneumonia-associated respiratory illness along with nebulization if required (Drożdżal et al., 2020). On the other hand, it was observed that the use of both non-aspirin NSAIDs and COX-2 inhibitors such as celecoxib might be associated with an augment in cardiovascular risk. Therefore, these drugs need to be further evaluated in COVID-19 patients with cardiovascular disease (Thomas et al., 2017).

## 8 Lessons Learned from Drug Toxicity During the Clinical Trials or Drug Repurposing Studies for COVID-19 Drugs

Thousands of clinical trials are being performed to understand the safety and efficacy of the repurposed drugs for COVID-19. Some drugs have shown drug toxicity during clinical trials (Chary et al., 2020). It has been noted that the use of psychotropic medicines might show toxicity to COVID-19 patients. Therefore, the dosing of psychotropic drugs is a critical factor and, continuous monitoring is necessary for some drugs like valproate, lithium, clozapine, etc. (Sabe et al., 2021). Several studies have reported hydroxychloroquine and chloroquine toxicity; specifically, cardiovascular toxicity has been observed in several cases (Kochi et al., 2020; Stevenson et al., 2020). Drug-drug interaction is a problem for comorbid COVID-19 patients, which might generate toxicity. It has been noted that anti-SARS CoV-2 drug candidates might have interactions with hepatic transporters. Therefore, this can cause liver toxicity. Many interactions were reported related to this issue (Ambrus et al., 2021). Drug toxicities related to the drug-drug interaction should be monitored appropriately. Moreover, other drug toxicities should be accurately observed to understand the toxicity profile of newly repurposed drug candidates.

## 9 Lessons Learned from Computational Drug Repurposing for COVID-19: Another Effort for Drug Discovery of COVID-19

Repurposing the drug is a fast way to identify new uses of existing medicines with a well-established safety and pharmacological profile. In this direction, thousands of molecules can be evaluated through different steps such as high-throughput screening and in silico approaches, which are lower-cost and faster screening processes. The computational drug repurposing can be an initial process and the filtering step (Park, 2019). Several researchers tried to identify the repurposed drugs for COVID-19 through this process. Therefore, computational drug repurposing is a prominent area for the pandemic period of COVID-19 (Galindez et al., 2021; Luo et al., 2021; Singh and Villoutreix, 2021; Srivastava and Singh, 2021).

### 9.1 Some Significant Approaches

In this direction, different repurposed approaches were utilized, such as phenotypic screening, binding assay, drug-centric approach, target-based approach, knowledge-based approach, and signature-based approaches (Parvathaneni and Gupta, 2020). However, several studies use a drug-centric method where FDA-approved drugs were taken into consideration. At the same time, a target-based approach was also used where protein drug targets were taken as a significant consideration. Other than these approaches, molecular docking, structure-based ML (machine-learning) strategies, network-based approaches, and hybrid methodologies have also been applied for computational drug repurposing for COVID-19 (Aronskyy et al., 2021).

### 9.2 Some Significant Studies

It has been reported that several protein drug targets have been indented, and the 3D structure of the protein target was generated from time to time. The 3D form was used to identify the new drug development of COVID-19 (Chakraborty et al., 2021d), and used for computational drug repurposing. It has been noted that many studies were performed using 3CLpro for protein drug targets. In this direction, more than 17 studies focused on predicting the repurposed drug-using 3CLpro for COVID-19 (Galindez et al., 2021). Other than the 3CLpro, several researchers have searched drug compounds using PLPro and RdRp for computational drug repurposing for COVID-19. A recent study by Chen et al. explored drug molecules of the combined databases such as DrugBank and KEGG databases. The study screened 7173 drugs and proposed 16 drug candidates for future studies (Chen YW. et al., 2020).

Similarly, Li et al. tried to analyze differential gene expression related to COVID-19-activated pneumonia using transcriptomic signatures. They have also tried to elicit the mechanism of cytokine storm syndrome during the acute phase of infection of COVID-19. The study concluded that some anti-inflammatory therapeutics might have beneficial effects, such as rituximab, tocilizumab, and adalimumab. The therapeutics may also have positive effects to restore the standard transcriptome in COVID-19 patients (Li et al., 2021). Using crystal of main protease structure, Wang has identified some drug candidates such as Carfilzomib, a bioactive compound (PubChem 23727975) (Wang, 2020). Another recent study was performed by Mahmoud et al. using DAAs (direct-acting antivirals). In this study, 16 FDA-approved DAAs were used to treat the hepatitis C virus (HCV). The study predicts their effectiveness through computational drug repurposing using SARS-CoV-2 drug target Mpro (main protease) and human targets such as hACE2 and human cathepsin L. They found Telaprevir as a good candidate, which they further evaluated *in vitro* (Mahmoud et al., 2021). Simultaneously, through the deep-learning method, Zeng et al. identified 41 repurposed drugs, including indomethacin, dexamethasone, etc. The study includes different open data resources and builds 15 million edges which contain 39 types of parameters (diseases, drugs, expression, pathways, proteins/genes, etc.). A connection of relationships was retrieved from the scientific literature (24 million) using the PubMed database (Zeng et al., 2020).

### 9.3 Identified Drug Candidates Through Bioinformatics/Computational Drug Repurposing

It has been noted that a good number of candidates have been identified through computational drug repurposing for COVID-19 patients. In general, it has been noted that mainly two categories of therapeutic candidates have been identified, which are either chemical entities or therapeutic antibodies. In the first category, the identified candidates are remdesivir, favipiravir, oseltamivir, dexamethasone, ivermectin, etc. (Wang and Guan, 2021). In the second category, the identified candidates are rituximab, tocilizumab, adalimumab, etc. (Li et al., 2021).

### 9.4 A Unified Strategy is Necessary

For COVID-19 computational drug repurposing, researchers have used different approaches, such as several types of computational tools and several sets of drug candidates. However, it is necessary to fix a unified strategy. At the same time, a validation strategy is also required in this direction.

## 10 Conclusion

To fight against the COVID-19 pandemic, several existing drugs have been repurposed to evaluate the treatment of COVID-19 patients (Table 2) (Saha RP. et al., 2020). Several clinical trials have been initiated in this direction. Until March 2021, 4952 clinical trials have been registered in ClinicalTrials.gov toward drug and vaccine development for COVID-19. It was observed that more than 100 countries have participated in contributing to these clinical trials. Other than the registered clinical trials (medium to large-size), several small-size clinical trials have also been conducted from time to time to evaluate the treatment of COVID-19.

Conversely, in addition to single-molecule therapy, several combination therapies were also evaluated to understand the treatment of COVID-19 patients. It was revealed from the significant clinical trials that remdesivir, tocilizumab, dexamethasone, and baricitinib might impart some beneficial outcomes for COVID-19 patients. On the other hand, the combination therapy of remdesivir and baricitinib also demonstrated better results than remdesivir alone for COVID-19 patients. We have learned from the previous experiences that the short-term repurposing of existing drugs might not provide a successful outcome for COVID-19 patients. Therefore, more clinical trials should be initiated to search for better therapeutics for COVID-19 patients using potential anti-viral molecules, being evaluated in different phases of clinical trials. Recently Professor Peter Horby (at the University of Oxford) has expressed the same view during a discussion with (Mullard, 2021). In affirmation, Dr. Collins, director of the NIH, wrote an editorial in *Science Magazine* and expressed the same view (Collins, 2021).

The medical science fraternity is trying to understand the proper treatment regimens for severe COVID-19 patients and solve the problem shortly. Another lesson learned has been the incomparable global collaboration spirit for drug and vaccine development. Different collaborations have been formed to resolve the health crisis since scientists have called for cooperation and partnership to stop the pandemic (Sanyaolu et al., 2020). The elderly with comorbidities such as diabetes, cardiovascular disease, immunosuppression-related diseases, and cancer are more susceptible to COVID-19 and are at greater risk (Saha RP. et al., 2020) since the high mortality rate has been noted from this group.

The vaccine is the best choice to contain this pandemic, and therefore the vaccination programs have been started globally. Billions of dollars are being spent on vaccine development efforts to find an effective vaccine for the infection, and many countries have contributed funds for the vaccine development. Several valuable lessons have been learned from the crisis. Once Bill Gates asked for a wake-up call after the Ebola epidemic and suggested preparing for a future pandemic, published in the NEJM (Gates, 2015). This COVID-19 pandemic also asks for a wake-up call for the next pandemic, and the lessons we have learned thus far will help to control future pandemics in a better way.
